# Genetic analyses favour an ancient and natural origin of elephants on Borneo

**DOI:** 10.1038/s41598-017-17042-5

**Published:** 2018-01-17

**Authors:** Reeta Sharma, Benoit Goossens, Rasmus Heller, Rita Rasteiro, Nurzhafarina Othman, Michael W. Bruford, Lounès Chikhi

**Affiliations:** 1 Instituto Gulbenkian de Ciência, Rua da Quinta Grande, 6, P-2780-156, Oeiras, Portugal; 2 Organisms and Environment Division, School of Biosciences, Cardiff University, Sir Martin Evans Building, Museum Avenue, Cardiff, CF10 3AX UK; 3 Danau Girang Field Centre, c/o Sabah Wildlife Department, Wisma Muis, 88100 Kota Kinabalu, Sabah Malaysia; 4 Sabah Wildlife Department, Wisma Muis, 88100 Kota Kinabalu, Sabah Malaysia; 5 Sustainable Places Research Institute, Cardiff University, 33 Park Place, Cardiff, CF10 3BA UK; 6 Department of Biology, University of Copenhagen, Universitetsparken 15, DK-2100 Copenhagen Ø, Denmark; 7 Department of Genetics and Genome Biology, University of Leicester, Adrian Building, University Road, Leicester, LE1 7RH United Kingdom; 8 School of Biological Sciences, University of Bristol, Life Sciences Building, 24 Tyndall Avenue, Bristol, BS8 1TQ UK; 9 CNRS, Université Paul Sabatier, ENFA, UMR 5174 EDB (Laboratoire Evolution & Diversité Biologique), 118 route de Narbonne, F-31062 Toulouse, France; 10 Université Paul Sabatier, UMR 5174 EDB, F-31062 Toulouse, France

## Abstract

The origin of the elephant on the island of Borneo remains elusive. Research has suggested two alternative hypotheses: the Bornean elephant stems either from a recent introduction in the 17th century or from an ancient colonization several hundreds of thousands years ago. Lack of elephant fossils has been interpreted as evidence for a very recent introduction, whereas mtDNA divergence from other Asian elephants has been argued to favor an ancient colonization. We investigated the demographic history of Bornean elephants using full-likelihood and approximate Bayesian computation analyses. Our results are at odds with both the recent and ancient colonization hypotheses, and favour a third intermediate scenario. We find that genetic data favour a scenario in which Bornean elephants experienced a bottleneck during the last glacial period, possibly as a consequence of the colonization of Borneo, and from which it has slowly recovered since. Altogether the data support a natural colonization of Bornean elephants at a time when large terrestrial mammals could colonise from the Sunda shelf when sea levels were much lower. Our results are important not only in understanding the unique history of the colonization of Borneo by elephants, but also for their long-term conservation.

## Introduction

The Bornean elephant (*Elephas maximus borneensis*) is one of four recognized subspecies of Asian elephant and is morphologically and behaviourally distinct from the elephants of mainland Asia^1^. It is classified as endangered according to the IUCN (International Union for Conservation of Nature) Red list of threatened species.

The origin of elephants on Borneo has been controversial and the subject of intense debate. Two competing hypotheses have been proposed: one suggests that elephants are non-native to Borneo and were historically introduced and the other that they are indigenous to the island. The introduction hypothesis is based on historical records suggesting that the current population represents the descendants of a domesticated herd that formerly existed on Sulu Island, Philippines, and were introduced to eastern Sabah by the Sultan of Sulu in the 17^th^ Century^1–3^. The original founders of these elephants most likely came from the Javan elephant population, which is now extinct^3,4^. It was further reported that only two elephants were introduced to Jolo (Sulu) Island in the late 13^th^ century, and their descendants were transported to Sabah around 1673^1,3^. Historical records fail to show exactly when and how many elephants the Sultan of Sulu translocated to eastern Sabah, but the current interpretation of the scenario involves a few individuals^3^. This hypothesis hence suggests two recent and successive founder effects during the history of the Bornean elephant, corresponding to the introduction of elephants from Java to Sulu and the introduction of the descendants of the Sulu stock to Borneo. Taken together, the introduction hypothesis implies that the current Bornean elephant population descends from a very low number of individuals 300–330 years ago, and a low genetic diversity in these founders must be assumed due to the recent founding of the Sulu population. Using a generation time of *ca*. 15 years this would correspond to *ca*. 20 generations ago.

The competing hypothesis argues that the Bornean elephant is indigenous to Borneo and that—despite the possible introduction of domestic elephants to Borneo—the wild population was not introduced by humans. This hypothesis gained prominence after a landmark study demonstrated the genetic distinctiveness of the Bornean elephant and its derivation from a Sundaic stock^5^. Interestingly, the single mitochondrial (mtDNA) haplotype found in the Bornean elephant was not observed in any of the extant Asian elephant populations from South and Southeast Asia, although no samples from the extinct Javan elephant have been sequenced to date. Time estimates, based on mtDNA sequence divergence, suggested a separation of the Bornean elephant haplotype and its closest relative from a common ancestor around 300,000 years ago. On that basis, the authors concluded that extant Bornean elephants are derived from endemic (Pleistocene) progenitors. In contrast, Cranbrook *et al*.^1^ have argued that this distinctiveness could also be exhibited if the present population comprises descendants of the (now extinct) Javan elephant. The absence of direct evidence lends support to the introduction hypothesis: there has been no confirmed finds of Asian elephant fossils in any excavation, including those in the Niah and Madai caves (within and outside the current species range in the Malaysian State of Sabah, respectively) although other large ungulates, such as Javan rhinoceros (*Rhinoceros sondaicus*), and Malayan Tapir (*Tapirus indicus*) are present^6–8^. Additionally, if the elephant had been on Borneo for tens of thousands of years its current limited geographic distribution in northern Borneo – which is not proximate to known Sundaland land bridge points on Borneo – could be considered surprising.

The genetic distinctiveness of the Bornean elephant from other mainland Asian elephant subspecies makes it one of the highest priority populations for Asian elephant conservation^5^. The lack of variation at the mtDNA control region is consistent with low levels of genetic diversity observed in nuclear genetic markers, including microsatellites and single nucleotide polymorphisms for this species^5,9,10^. It has been suggested that either i) the occurrence of recent founder events^1^ and/or ii) the persistence of a low population size primarily due to postglacial colonisation are responsible for such reduced genetic variability^5^.

Identification of species introduction events, pathways and putative source populations has traditionally been accomplished by examining archaeological evidence and recorded dates of first discovery into introduced areas, and increasingly by using genetic analyses of native and introduced populations^11,12^. Population genetics has provided useful approaches to resolve the native *versus* introduced status of a species, reconstruct the routes and origin of these introductions, and elucidate colonization histories^13,14^. Approximate Bayesian computation (ABC) makes use of simulations and likelihood-free inference to contrast the complex demographic models commonly used, and has proven useful in reconstructing the demography of many species, including humans^15–19^. In the present study, we employed both a full-likelihood Bayesian approach (i.e. the MSVAR method) and ABC to compare different approaches and to estimate demographic and historical parameters including founding effective population size and introduction times using microsatellite and mitochondrial data. We more specifically aimed to test competing models of the demographic history of the Bornean elephant. Their limited distribution, enigmatic origins, and critical conservation status suggest that knowledge of their past population demography would be useful for the development of a sound conservation strategy.

## Results

### Quantifying changes in effective population size and dating with MSVAR

**Figure 1:**
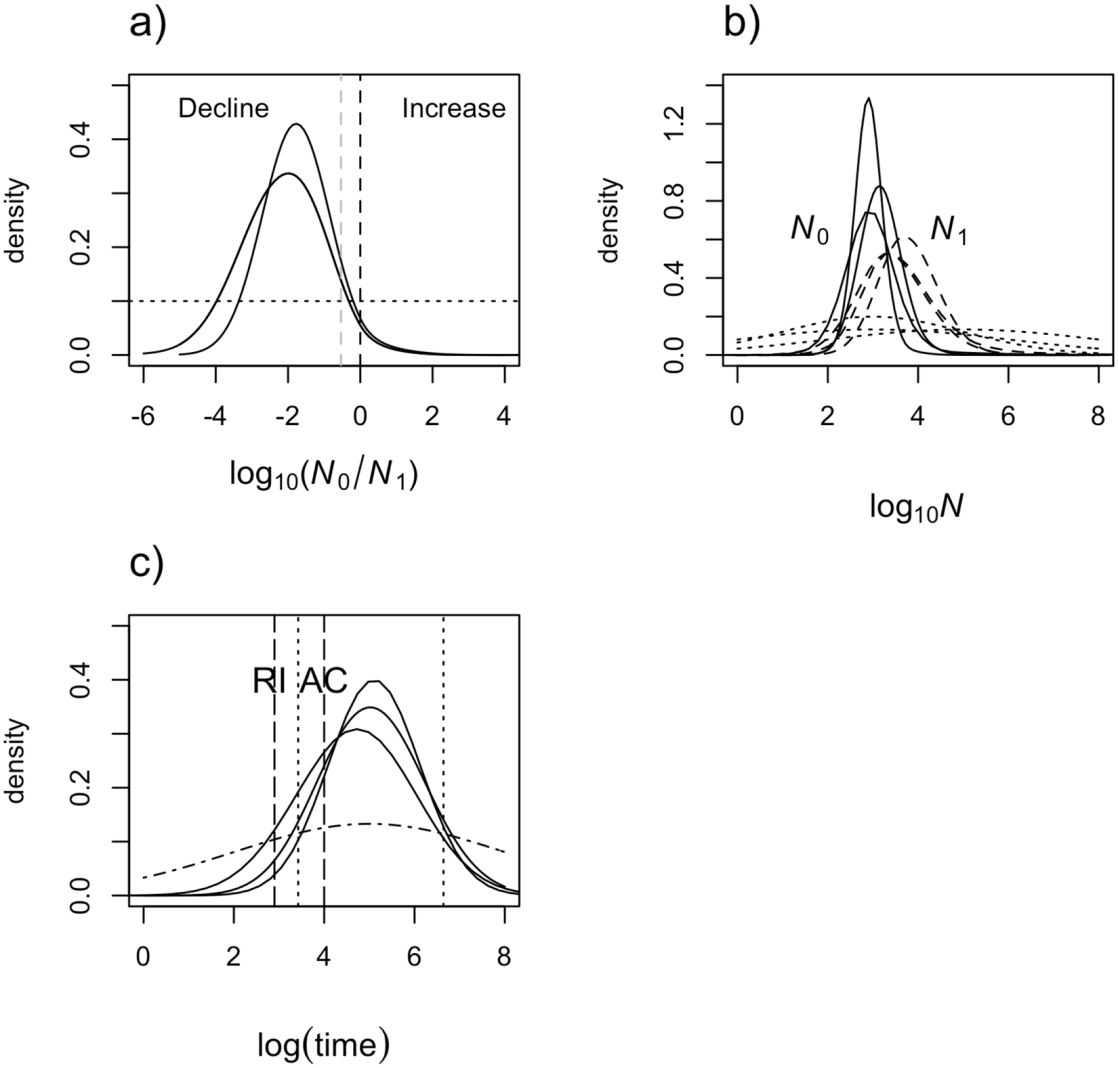
MSVAR estimates of changes in effective population size, quantification and timing of a population bottleneck in the Bornean elephant population. Note that for illustrative purposes, only the plot for the pooled Bornean elephant samples is shown (population-level results are presented in Figure S1); (**a**) The log_10_ ratio of current to ancestral population size (N_0_/N_1_). Solid lines (two independent runs) correspond to the exponential population size change model. The dotted black and grey vertical lines corresponds to the absence of population size change, log (N_0_/N_1_) = 0 and 95% quantile of the posterior distribution, respectively. The prior distribution is shown for comparison (flat dotted line), (**b**) Posterior distributions of the current (N_0_ in thick lines) and ancestral (N_1_ in dashed lines) effective population size using an exponential model. Different curves correspond to the posterior distribution obtained by independent MCMC runs. Dotted lines correspond to the different priors used for N_0_ and N_1_, (**c**) The posterior distribution of the time since population bottleneck is represented on a logarithmic scale. The different black vertical long dashed lines correspond to recent introduction (RI) and ancient colonization (AC), respectively. There is no evidence of a population bottleneck closer to the period of recent introduction (i.e. 300 years ago). The most extreme 5% and 95% quantile of the posterior distribution are shown as black dotted lines. The prior is shown as dot-dashed line, its median being 100,000 years ago.

We investigated whether the genetic data were consistent with a very recent introduction of *E*. *maximus* into Borneo 300 years ago, followed by a large population expansion, as assumed on the basis of historical records. We conducted MSVAR analyses on a data set constructed by resampling 35 individuals randomly, first in the Bornean elephant sample as a whole, and then within each sampled population separately. We found that the genetic data exhibited a signal consistent with a population decline whether we assumed a linear or an exponential model for population size change (Fig. 1). Population-level results are presented in the supporting materials. The median value from pooled posterior distributions of log_10_ (*N*_0_/*N*_1_) was −1.76 (90% HPD = −4.24/−0.53), corresponding to a greater than 50-fold population decline. Coalescent analyses on each population from different locations in Sabah taken separately revealed the same signature of population decline (Fig. S1). Population structure can bias demographic inference by creating a spurious signal of population size change in ways that depend on the sampling scheme^20^. However, in this case, the similarity of the results under two different sampling schemes makes it less likely that the decline signal is an artefact. This is further supported by the similarity of the decline signal detected using the ABC approach under the AC and ACS models (where AC and ACS correspond to Ancient colonization scenarios, without and with population structure, respectively; see below). The estimates of current (median *N*_0_) and ancestral population size (median *N*_1_) for the pooled data were 833 (90% HPD = 143/4,766) and 4,820 (90% HPD = 753/53,154), respectively. Estimates of the time of population contraction showed support for population decline occurring long before the recent introduction of elephants to Borneo (median *T* = 57,000 years before present or ≥3,800 generations ago). While the estimated 90% HPD limits were broad, ranging between 1,000 and 1,311,000 years, the most recent estimate still predates the alternative hypothesis that suggests an introduction 300 years ago.

### Quantifying changes in effective population size and dating using an Approximate Bayesian Computation (ABC) approach

(i) Microsatellite coalescent simulations

#### Comparison of alternative demographic models

**Figure 2:**
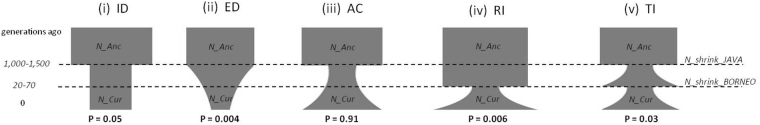
Demographic models investigated in this study and their posterior probabilities (P). Parameters estimated are indicated in italics (*N_Anc*: ancestral effective population size, *N_Cur*: current effective population size, and *N_shrink*: effective number of individuals at the time of bottleneck). Abbreviations of demographic models: (**i**) instantaneous decline (ID), (**ii**) exponential decline (ED), (**iii**) ancient colonization (AC), (**iv**) recent introduction from Sulu/Java (RI), and (**v**) two introductions (TI). Further details in the supporting materials.

Our ABC framework allowed discrimination among five simple (non-structured) models (Table 1 and Fig. 2). Posterior probabilities clearly rejected models assuming a recent introduction from Sulu and Java (i.e. RI and TI models) as well as models assuming no founder events. For 10,000 of the retained simulations (1% of the total number), the strongest support was obtained for the AC (i.e. the *ancient colonization*) model with a posterior probability greater than 0.91 (using multinomial logistic regression). The posterior probability of this model for the 1,000 closest simulations was 0.93. This model assumes an ancient colonization of elephants into Borneo accompanied by a founder event. The models emulating a recent introduction (RI and TI) had a posterior probability of 0.006 and 0.03 (Table 1). When we tested the robustness of our inferences on demographic parameters using different priors for the simple set of models, the posterior probability indicated that under all prior sets the most supported model was the AC model.

**Table 1:** Approximate Bayesian computation model choice analysis.

Tolerance	Simple models				
	ID	ED	AC	RI	TI
rejection					
0.001	0.16 (4.12)	0.04 (17.04)	0.70	0.08 (8.71)	0.06 (10.39)
0.01	0.22 (2.11)	0.08 (5.55)	0.45	0.16 (2.86)	0.09 (5.06)
regression					
0.001	0.03 (29.56)	0.01 (84.39)	0.93	0.01 (75.71)	0.05 (19.65)
0.01	0.05 (18.14)	0.004 (247.5)	0.91	0.006 (142.2)	0.03 (36.44)

Model comparison of the simple models. Posterior probability values for each model and Bayes factors in brackets (of the best supported model against the respective models) are shown for the rejection and multinomial logistic regression methods and for different values of tolerance. Abbreviations of demographic models: instantaneous decline (ID), exponential decline (ED), ancient colonization (AC), recent introduction from Sulu/Java (RI), two introductions (TI).

When model selection was performed on all models, including the two structured models (i.e. ACS and RIS), the ACS model exhibited the highest support followed by the AC model (Table 2). In addition, parameter estimates obtained for the AC model were almost identical to those obtained with ACS (Table 3). The marginal densities of the AC and ACS models using *ABCtoolbox* had *P* values above 0.6 with the ACS model being more likely (Supplementary Table S1). This indicates that the observed summary statistics are within the range of the marginal densities for the retained simulations and that the models were thus capable of producing the observed summary statistics. In conclusion, the ABC results are at odds with a recent human introduction of elephants in Borneo. They strongly favor a more ancient founder event, after which subsequent population structure or fragmentation may have also been important (model AC *versus* ACS). This is in agreement with the recent work of Goossens *et al*.^10^ which identified significant genetic differentiation among fragmented populations.

**Table 2:** Approximate Bayesian computation model choice analysis.

Tolerance	Simple and structured models						
	ID	ED	AC	ACS	RI	RIS	TI
rejection							
0.001	0.03 (16.68)	0.01 (40.49)	0.40 (1.30)	0.52	0.01 (36.00)	0.001 (363.7)	0.02 (23.61)
0.01	0.13 (3.13)	0.02 (21.39)	0.34 (1.21)	0.41	0.04 (11.17)	0.02 (573.2)	0.04 (9.67)
regression							
0.001	0.01 (49.67)	0.01 (46.90)	0.41 (1.35)	0.56	0.009 (59.35)	0.0002 (1297)	0.02 (35.95)
0.01	0.009 (61.04)	0.007 (81.84)	0.41 (1.42)	0.57	0.006 (88.92)	0.0001 (4211)	0.009 (61.08)

Model comparison of the simple models including the two structured models. Posterior probability values for each model and bayes factors in brackets (of the best supported model against the respective models) are shown for the rejection and multinomial logistic regression methods and for different values of tolerance. Abbreviations of demographic models: instantaneous decline (ID), exponential decline (ED), ancient colonization (AC), recent introduction from Sulu/Java (RI), two introductions (TI), ancient colonization split (ACS) and recent introduction from Sulu/Java-split (RIS).

**Table 3:** Prior and posteriors parameter estimates for AC, RI, ACS and RIS models.

Parameter	Interpretation	Prior	Posterior				
		type [min,max]	5%	mean	median	mode	95% HPD
**ancient colonization (AC)**							
N_Anc	Ancestral effective population size	loguniform [4,5]	10,747	33,135	32,681	54,262	87,096
N_Cur	Current effective population size	loguniform [2.4,3]	257	485	480	308	969
T_shrink	time of bottleneck	uniform [1000,1500]	1,018	1,220	1,207	1,242	1,463
N_shrink	effective number of individuals introduced	uniform [4,50]	5	28	29	45	49
**recent introduction from Sulu/Java (RI)**							
N_Anc	Ancestral effective population size	loguniform [2.4,4]	602	2,032	1,971	1,128	6,918
N_Cur	Current effective population size	loguniform [2.4,3]	245	470	448	286	977
T_shrink	time of bottleneck	uniform [20,70]	24	44	46	47	69
N_shrink	effective number of individuals introduced	uniform [2,50]	7	25	23	15	47
**ancient colonization-split (ACS)**							
N_Anc	Ancestral effective population size	loguniform [4,5]	10,000	30,902	31,325	31,622	89,125
N_Cur	Current effective population size	loguniform [2.4,3]	251	489	498	295	977
T_shrink	time of bottleneck	uniform [1000,1500]	1,020	1,233	1,224	1,176	1,471
N_shrink	effective number of individuals introduced	uniform [8,50]	9	28	28	23	49
**recent introduction from Sulu/Java-split (RIS)**							
N_Anc	Ancestral effective population size	loguniform [2.4,4]	512	691	660	616	1,071
N_Cur	Current effective population size	loguniform [2.4,3]	501	812	851	933	977
T_shrink	time of bottleneck	uniform [20,70]	10	61	64	67	69
N_shrink	effective number of individuals introduced	uniform [8,50]	40	32	31	46	50

Population size parameters are in units of population effective size (Ne), while time parameters are in units of generations. Mean mutation rate (μ) is same in all the models. The prior values for the mutation rate were 10^−5^ to 10^−3^. Note that the posterior estimate values were converted from log to linear scale.

#### Validation of model selection and parameter estimation

When comparing all five simple models simultaneously, the cross-validation method was not able to discriminate them. The ED and AC models were consistently easiest to identify. However, in the pairwise model comparisons, and in the simultaneous evaluation of the four models (i.e. AC and RI models with and without structure), cross-validation support improved substantially (Supplementary Tables S2a,b and c). In the pairwise comparisons, all models were identified accurately when compared to the AC model. Cross-validation result for the four models indicates that ABC clearly discriminated between the AC/ACS and RI/RIS models. They were classified correctly 413, 595, 451 and 727 times out of 1000 (Supplementary Table S2c). However, the method was not able to distinguish between the simple and the structured version of the same model (i.e. between AC, and ACS, RI and RIS), which could be because these models (with and without structure) are very similar. Altogether, this cross-validation provided confidence in the model choice results and further support to the high posterior probability values obtained for the AC model using the model choice procedure.

**Figure 3:**
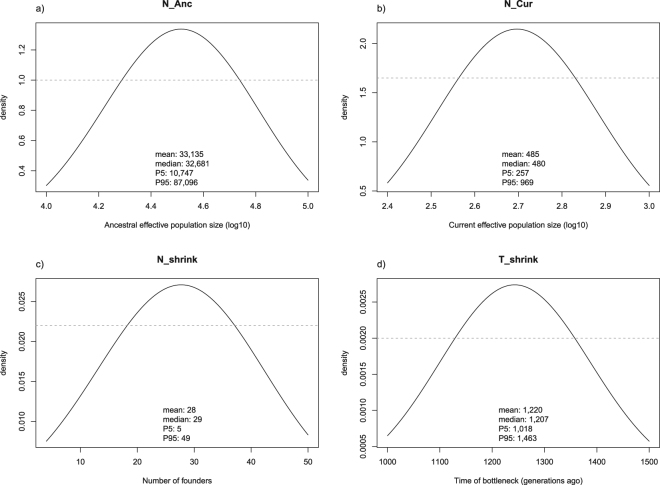
Posterior (black solid lines) and prior (dashed grey lines) distributions of demographic parameters for Bornean elephant estimated through ABC analysis and according to the best-fitting model (ancient colonization). Point estimates and 95% credibility intervals for all key parameters obtained through simulations are also given. Note that in (**a**) and (**b**) values were converted from log to linear scale. See Fig. 2 and Table 3 for further details on the demographic parameters.

We estimated the posterior distributions for the parameters in the best-supported model (i.e. the AC model with wide prior on mutation rate for all microsatellite loci; Table 3, Fig. 3). Under this model we could infer that the Bornean elephant population was founded by a small population with an effective size of ~28 individuals [95% highest probability density interval (HPD): (5–49)]. This founder population came from a larger ancestral population with estimated effective size of 33,000 individuals [95% HPD: (10,747–87,096)]. The founder population gradually expanded to its current effective population size of 485 individuals [95% HPD: (257–969)]. We estimated that this expansion dates to around 18,300 [95% HPD: (15,270–22,000)] years before present. Figure 3 shows that the posterior density curves of the current (*N_Cur*), and ancestral effective population size (*N_Anc*), differ noticeably from the priors. This indicates that these posterior values were informative as being derived from the genetic data rather than being driven by the prior values.

#### Examining the influence of prior assumptions

For our initial ABC simulations, we chose prior distributions tightly bounded around values based on the available historical data. However, to explore sensitivity to these assumptions, we conducted additional simulations with broader prior assumptions on effective population sizes and the time of bottleneck (see supporting materials). In all cases, the same demographic model (AC) was the most supported as in the initial simulations. For comparison, we note that the supplementary simulations based on fixed mutation rate value (i.e. 10^−3^) and broader prior assumptions on *N_Anc* and *T_shrink* yielded lower posterior estimates than in our initial simulations. Notably, the time (*T_shrink*) in this model exhibits a posterior which was again different from the prior with a mean estimate of 11,400 years before present. Additionally, the *T_shrink* parameter was insensitive to the mutation rate, whereas, as expected, the ancestral and current population sizes were (see Fig. S6). Therefore, the dating of the founder event in the AC scenario is robust and relatively ancient. This is not necessarily as surprising as it may seem since genetic diversity after a bottleneck event is mainly driven by genetic drift, and does not depend so much on mutation rates.

We further tested the impact of mutation rate on our inferences by simulating the AC and RI demographic models a second time, using fixed mutation rates of 10^−3^, 10^−4^, and 10^−5^. Simulation of the two models using different mutation values still favoured the AC model (with mean mutation rate 10^−3^ for all loci) over RI and revealed similar estimates for the dating of the founder event (18,000 years before present, Fig. S7).

(ii) mtDNA simulations

The results from the simulations using mtDNA data showed the AC model to have the highest number of simulations (64,344 out of 100,000) with zero diversity, as observed in present-day Bornean elephants, consistent with the results obtained using microsatellite data (Supplementary Table S5). However, two other models (RI and TI) also yielded a high proportion of zero diversity (49,451 and 54,564), making it difficult to distinguish the three models (AC, RI and TI) using mtDNA data alone.

## Discussion

The present study focused on the past demographic history of the Bornean elephant over multiple timescales using two complementary approaches. The current consensus is that elephants were anthropogenically introduced to Borneo during the 17th century^1,3^. However, Fernando *et al*.^5^ suggested a different scenario. Using divergence of mtDNA haplotypes, and the absence of a source population on the mainland, they concluded that the Bornean elephant might have been present in Borneo for as much as 300,000 years. The pervasive argument against this hypothesis is that Pleistocene or Holocene remains of the elephant have never been found in this region^21^.

Our results suggest that neither of these hypotheses are the most likely given the genetic diversity in the current population. Although our results do not enable us to pinpoint the precise time of colonization, we find that the best-fitting scenario involves an end-of-Pleistocene bottleneck, probably between 11,000–18,000 years ago, roughly coinciding with the end of the Last Glacial Maximum (LGM). Our results do not support a substantial bottleneck in more recent times, and this would seem to refute the literal version of the introduction scenario. As an illustration of this, the allelic richness observed in the Sabah population is incompatible with a low number of recent founders (Fig. S8). Our findings do not rule out another founder event predating the LGM bottleneck, because our data do not allow us to resolve the history beyond this point. Therefore, we cannot conclusively exclude a more ancient origin than the bottleneck found here. Our analyses, however, provide strong support for a late Pleistocene founder effect followed by a gradual population expansion. This scenario is in agreement with a natural colonization of Borneo at the time of the last land bridge between the Sunda Islands^22,23^. Additionally, our simulations suggest that the standing genetic diversity would be reduced to levels similar to that observed, if the elephant population remained small for a thousand generations. In other words, our data could fit the scenario suggested by Fernando *et al*.^5^ but this would require a very long time during which the population remained small and isolated.

Several historical parameters help to further define the favored AC scenario. Differences between the posterior and prior density curves for time of bottleneck event (*T_shrink*) were conspicuous with unimodal posteriors, indicating that the genetic data are informative for this parameter. Moreover, additional analyses showed that when we allow for very recent events in our priors, the posterior distributions always support the ancient event (the *AC model with extended priors*, see supporting materials). Parameter estimates obtained for simulations exploring prior boundaries on the time of bottleneck were in general highly congruent.

Support for the selected model (AC) was high in all analyses performed and was robust to different prior assumptions. Cross-validation based on simulated pseudo-observed datasets, however, revealed only moderate identifiability of the models when all five models were examined at once. This could indicate the limited power of the summary statistics and/or that the models differ in their specificity - e.g. some models encompass a much larger demographic parameter space than others. However, pairwise model comparisons supported our choice of AC as the leading model of colonization history and suggested that this model was likely to be preferred when it was true and unlikely to be preferred when it was false. Thus, among the models considered, the ancient colonization scenario is the best-fitting model of the Bornean elephant’s colonization history.

Based on the founder effect inferred during the colonization of Borneo and assuming that Bornean elephants have had a relatively low population size, we show that the genetic diversity should indeed be low compared to that of other Asian elephants. Elephant populations in Borneo do exhibit reduced genetic diversity in comparison with the mainland Asian populations coupled with high genetic differentiation from all mainland populations, implying a strong effect of genetic drift following the colonization of Borneo^5,10^. While an ancient bottleneck would have reduced the genetic variation in the Bornean elephant, social behaviour and in particular strict maternal philopatry also leads to structured genotypic variation at the population level^24^. Our results suggest that the Bornean elephant population is most likely to have experienced a strong bottleneck or founder event involving few individuals only, a situation supported by its mtDNA monomorphism^5,10^. It is entirely plausible that all but one mtDNA haplotype could have been lost due to founder effect followed by genetic drift, which our simulations also support^25^. Further, we confirmed by simulation that the presence of a single mtDNA haplotype is no less likely under the ancient than under the recent introduction scenario (Supplementary Table S5).

The lack of elephant fossils on Borneo has often been taken as a strong argument that it cannot be indigenous to the island^21^. Remains of other large mammal fauna, such as orang-utan (*Pongo pygmaeus*), Javan rhinoceros (*Rhinoceros sondaicus*), etc. have been recovered from the Niah and Madai caves in northwest and north Borneo, proving their late Pleistocene and Holocene presence on the island^21,26^. However, there could be at least three reasons why elephant fossils have not been discovered in fossil sites on Borneo (but see^21,27^ for an account of possible elephant fossils found on Borneo). First, it might be that elephants did not yet occur on what is now Borneo during the time at which conditions were favourable for fossil deposition at known sites (due to their end-of-Pleistocene arrival as suggested in our scenario). Second, it might be that elephant fossils have simply not been found due to the poor preservation conditions in humid tropical areas and the fact that there are only a limited number of Late Pleistocene or Holocene fossil sites identified on Borneo^1,21,26^. If elephants colonized Borneo as late as during the LGM, only four of the recognised fossil sites on Borneo could possibly contain fossils, and the probability of encountering an elephant fossil here would be low if the Bornean elephant population expanded from a small founder number as suggested by the inferred AC model. Third, what we know about present-day Bornean elephants suggests a limited distribution and a small population. If this was also the case in the past it could explain the lack of fossils. However, we must also stress that the present distribution of Bornean elephants remains an unsolved puzzle. If Bornean elephants arrived from the Sunda shelf as our genetic data suggest, it is not clear why they are currently found in the region geographically most distant from where Borneo was connected to the Sunda shelf. Until fossil remains are found and dated, or more is understood about the ecological needs of Bornean elephants, the current geographical distribution of the Bornean elephants remains speculative. If elephants had been present in the island throughout the Pleistocene, one could expect them to have colonized the whole island as other large vertebrates have. For instance, orang-utans (that have been present in the island throughout the Pleistocene) are found throughout the island^28^. Likewise, the proboscis monkey (*Nasalis larvatus*), one of the largest monkey species in Asia, is endemic to the island of Borneo and found across all Borneo, where it also arrived via land bridge connections during the Pleistocene^29^. Interestingly, fossil records of proboscis monkey are also not known in Borneo^26,30,31^.

The demographic analyses that we performed using MSVAR showed results that are consistent but not in full agreement with the ABC results. In agreement with the ABC analyses, the MSVAR results did not support a recent population introduction. However, it suggested a much more ancient decline (around 3,500 generations ago) compared to the ABC estimates. MSVAR analyses suggested that the severity of decline varied and differed among the regions, and our ‘pooled’ results also showed an extensive range-wide population decline. This congruence across various sampling schemes suggests that the detection of a population decrease is not a simple artefact of population structure. However, our estimates have large confidence intervals and some of the assumptions of the model might be violated^20,32^ - notably the assumption of a single monotonic population size change and of a stepwise mutation model. The much more restricted model assumptions in MSVAR relative to our ABC analyses likely explain at least some of the discrepancies in estimates from the two methods—whereas they agree on the general trend of a larger ancestral than current population size, and they overlap in the timing of such an event. Indeed, when we included an MSVAR-like model in the ABC framework, we found low posterior support for this relative to the other ABC models (see supporting materials).

Based solely on contemporary genetic data, we show that the present-day genetic diversity of the Bornean elephant is best explained by a founder event that took place thousands of years ago. However, the geographic origin of the Bornean elephant is not resolved, as our demographic scenarios cannot distinguish between different geographic origins. It is possible that elephants could have dispersed naturally from Java rather than mainland Asia when the sea level was at least 40 m lower than at present. The major Sunda Islands (Java, Sumatra and Borneo) were connected several times via land bridges during periods of low sea levels in the late Pliocene and Pleistocene until the end of LGM about 10,000–15,000 years ago^22,33^. Therefore, many authors have characterized Sundaland as a geographical unit across which species should have been able to move freely during glacial periods until 10,000 years ago when higher sea levels started to separate the islands^34–36^. Rising sea levels in the Late Pleistocene may have isolated the small elephant population on Borneo, which since diverged from their source population. There is at present no possibility of discerning between a Java and an Asian origin of the Borneo elephant, as samples from the extinct Javan elephant have proven impossible to obtain. Furthermore, the elephant could have been already on Borneo before the LGM bottleneck identified here, in which case the bottleneck could simply be a demographic event associated with e.g. climatic factors or a within-island founder effect. However, this supposition is less compatible with the lack of Pleistocene fossils on the island.

Our conclusions should include some possible caveats. First, our ABC approach showed a moderate power to discriminate among the scenarios. This is probably a limitation of the resolution of microsatellite-derived summary statistics and is likely to improve once better genome-wide data can be obtained. Second, the demographic history of natural populations - particularly for endangered species with a complex history - is usually characterized by successive historical events, and the identification of multiple decline events can be statistically problematic^20,37^. Third, some factors can impair the ability of methodologies to detect a very recent bottleneck, in particular. This includes the possibility that a strong and ancient demographic event (e.g. a strong founder effect during the LGM) may be confounding the detection of more recent effective population size changes in Bornean elephant genome. Fourth, as has been suggested for long-lived species such as orang-utan, a long generation time could lead to retention of genetic diversity because of a shorter effective time of exposure to bottleneck compared to a species with short generation time^38^. This means that population size changes spanning the just 20 elephant generations that have occurred after a 17^th^ century introduction could theoretically be insufficient to drive the genetic signal. These caveats could be particularly biased against identifying a recent bottleneck in Bornean elephant, even if it had occurred. However, we also note that other studies^37,39,40^ have successfully found evidence for a recent demographic event from contemporary genetic data.

There are two further caveats to our observation that an ancient founding event is more in agreement with the data. First, we cannot exclude that there has been a continuous, human-mediated influx of elephants from other populations into the elephant population in Sabah in historical times^1^. This could lead to patterns of genetic diversity more in agreement with an ancient than a recent introduction scenario, even if the latter was true. However, there is no historical record of sustained elephant introductions to Sabah, and the recent introduction hypothesis is quite clear in positing a single introduction of just a few individuals^3^. Second, it should be noted that we only considered rather simplistic scenarios, and it is possible that a more complex scenario could better fit the observed data. However, the suite of scenarios considered here are reasonably likely to cover all the plausible general trends in the demographic history of the Bornean elephant.

In conclusion, this study shows that a recent anthropogenic introduction of the elephant to Borneo is less in agreement with genetic data than a natural origin. Since we identified a bottleneck most probably between 11,000 and 18,000 years ago - at the end of the Last Glacial Maximum where land bridges connected Sundaland - a likely interpretation is that the elephant colonized Borneo from another part of Sundaland at this time. We propose that this scenario should be given serious consideration when evaluating the natural history of the Bornean elephant, including its ecology, behaviour and conservation. Further research is needed to validate this hypothesis, including the use of more genetic data (such as sequencing the mtDNA from a Javan elephant fossil), but also palaeontological and biological studies of the Bornean elephant population.

## Methods

### Sample information

We used microsatellite data analysed in Goossens *et al*.^10^. This data set is derived from 224 individuals from across the range of the Bornean elephant genotyped at 18 microsatellite markers, with the addition of 630 bp of mtDNA region sequences from a subset of individuals (*n* = 60). Microsatellite data were available from four different locations in Sabah: (i) Lower Kinabatangan, (ii) North Kinabatangan range (Deramakot), (iii) multiple sites in the Central Forest (Ulu Segama-Malua, Gunung Rara, Kalabakan, Kuamut, Maliau Basin Conservation Area), and (iv) Tabin Wildlife Reserve as described in Goossens *et al*.^10^.

### Data analyses

#### MSVAR

i) Tests for changes in effective population size.

We investigated the demographic history of Bornean elephants, examining population size changes while accounting for population structure. We tested whether a signature of population decline is found in Bornean elephant populations and dated the occurrence of any such event. We used two different sampling schemes to investigate changes in effective size of Bornean elephant populations: (i) examining each sampling site, and (ii) examining a pooled (*n* = 35) sample set^20^.

Our analyses of population size changes were carried out using two different but complementary approaches (see Beaumont^41^, and Storz and Beaumont^39^) as in^37,38^. Both methods identify a single major change (increase or decrease), and quantify and date changes in effective population size. These are full-likelihood Bayesian methods that utilize the information from the full allelic distribution in a coalescent framework. We used the full data set with 18 microsatellite loci for both methods. Further details on the MSVAR analyses are provided in the supporting materials. For each dataset, we performed two independent runs with wide uninformative priors and a 15 year generation length^42^.

ii) Recent versus older population size changes

To separate recent from ancient factors, we further analysed the posterior distributions obtained for the time *T* since the population change (in generations). Additionally, we assessed the relative probability of recent *versus* ancient events by determining whether the data favoured events that were older or more recent than *T* = 53.3 generations. Assuming 15 years per generation this corresponds to 800 years. The value of 53.3 generations was chosen because it is very likely older than any historical record suggesting a movement of elephant from Sulu to Borneo. The corresponding value of 800 years is a likely conservative value since the generation time of elephants may be larger rather than smaller than 15 years.

#### Approximate Bayesian Computation (ABC) analysis

(i) Microsatellite coalescent simulations.

To infer the introduction history of elephants into Borneo, we used *fastsimcoal*^43^ to generate simulated microsatellite data under different demographic scenarios and *arlsumstat*^44^ to calculate summary statistics, coupled together in *ABCtoolbox*^45^. We defined and parameterized a number of potential colonisation models that constituted alternative hypotheses of demographic— and hence colonization, history — that have been proposed for the species. We kept demographic scenarios general and relatively simple and tested seven (5 simple and 2 complex) models in total (graphically depicted in Fig. 2, details in the Supporting materials). We set the number of loci and elephant sample sizes to those of the actual full dataset (224 individuals genotyped at 18 loci^10^), and microsatellite diversity was generated under the generalized stepwise mutational model (GSM) with some proportion of multistep mutations. In total, one million (10^6^) simulations were run for each model, and the 10,000 (1%) simulations with the smallest Euclidean distance were retained for ABC model choice testing and parameter estimation.

#### Model choice and parameter estimation

We compared the seven demographic models, which differed in the order of introduction and on whether founder events occurred. Following the selection of the datasets with the smallest Euclidean distance (*d*) to the observed summary statistics, we estimated the model fit and compared competing historical demographic models using two different methods. First, we used *ABCsampler* and *ABCestimator* from *ABCtoolbox* to calculate the marginal density for each of the retained 10,000 simulations and calculate a *p* value as the fraction of these that have a lower marginal density. The likelihoods were estimated for the models under General Linear Model post-sampling adjustment (ABC-GLM^46^). A low *p* value indicates that most retained simulations have higher marginal densities and suggests an inability of the model to produce the observed summary statistics. Second, we calculated the BF as the probability of one model *versus* another^45,47^.

We also used the ‘*abc*’ R package^48^ that implements several ABC algorithms for performing parameter estimation and model selection. To identify the most probable model, we computed the posterior probability of the five simple models above using a multinomial logistic regression approach on the retained simulations^49,50^. The *postpr* function was used to select the best model (estimate the posterior probability of each of the models). The ‘*abc*’ R package^48^ also includes a cross-validation tool for model selection, the accuracy of ABC estimates and also calculates the misclassification probabilities when performing model selection. For that, we randomly selected 1,000 datasets from the simulation output for each of the demographic model and used the function *cv4postpr* to assign a model to each of these datasets. Finally, we recorded the number of times that the true model was correctly identified (details on cross-validation is given in the Supporting materials). Parameter inference was performed using a local linear regression^15^ with a correction for heteroscedasticity^51^. We report the mean, median, mode and 95% highest posterior density (HPD) interval as an estimate of that population parameter.

(ii) Mitochondrial DNA (mtDNA) simulations

In a previous study^10^, we observed no mtDNA variability and a single mitochondrial haplotype in all Bornean elephant samples (*n* = 60) analysed from Sabah. We did not carry out a full ABC analysis with the mtDNA sequence data as monomorphic summary statistics across simulations preclude full ABC; however, we carried out simulations to evaluate how often each model yielded observations of zero mtDNA diversity. MtDNA data was simulated using priors drawn from the posteriors of the ABC results performed for the microsatellite data, with the *N*e scaled on female effective population size. We used the number of haplotypes (*H*), number of segregating sites (*S*) and average number of pairwise differences (*π*) within each of the statistical groups as summary statistics. We ran 100,000 simulations under each model and counted the proportion of simulations with zero diversity for each model. It was assumed that the model that was most likely to produce zero diversity was also the most likely one, although it must be emphasized that this is not a rigorous statistical test.

## Electronic supplementary material

Supporting materials
