# Supplementary material for: Genetic analyses favour an ancient and natural origin of elephants on Borneo
Source: Sci Rep. 2018 Jan 17;8:880. doi: 10.1038/s41598-017-17042-5 (PMC5772424; doi:10.1038/s41598-017-17042-5)
Supplement: Supplementary file 1 — Supporting materials [file 41598_2017_17042_MOESM1_ESM.pdf]

Supporting materials for "Genetic analyses favour an ancient and natural origin of elephants on Borneo"

Reeta Sharma, Benoit Goossens, Rasmus Heller, Rita Rasteiro, Nurzhafarina Othman, Michael W. Bruford, Lounès Chikhi

S1 Text: Microsatellite data analyses:
1. *MSVAR analyses:*
   1.1 Methods
   1.2 Further MSVAR results
2. *Approximate Bayesian computation (ABC) analyses:*
   2.1 Details on the main demographic models
   2.2 Structured models
   2.3 Summary statistics
   2.4 Validation of the model selection
   2.5 Goodness-of-fit
3. *Supplementary ABC simulations:*
   3.1 AC model with extended priors
   3.2 Effect of mutation rate
   3.3 AC model with equal priors on Ne
   3.4 MSVAR like model

S1 Text: Microsatellite data analyses

1. MSVAR analyses

1.1 Methods

The Beaumont method[1], implemented in the program MSVAR 0.4 assumes that a stable population of size N_1 (ancestral population size) started to decrease (or increase) T generations ago to the present-day population size (N_0). This method allows for either

linear or exponential changes in population size and to estimate the posterior probability distributions of (1) the magnitude of population size change, $r = N_0/N_1$, (2) the time since the population started changing size scaled by N_0, $t_f = t_a/N_0$, and (3) the scaled mutation rate $\theta = 2N_0\mu$, where μ is the per locus mutation rate per generation. For each sampled population, the analyses were performed under the exponential model using different parameter configurations, starting values and random seeds. In this study, wide uniform prior distributions were chosen (between -5 and 5 on a $\log_{10}$ scale) for $\log(r)$, $\log(\theta)$, and $\log(t_f)$. Positive $\log(r)$ values, corresponding to a population expansion, were set as the MCMC starting point. Although MSVAR 0.4 allows for quantification of a population increase or decrease, N_0 and N_1 cannot be estimated independently. Similarly, it can only approximate t_a as a time scaled by N_0, with N_0 remaining unknown. The total number of iterations was always larger than 5×10^9 with a thinning interval of 5×10^4. This method was employed first in order to test for a genetic bottleneck under different models of population size changes.

The Storz and Beaumont method[2] implemented in MSVAR 1.3 quantifies the effective population sizes, N_0, N_1 and the time T (in generations) since the population size started to change. This method assumes lognormal prior distributions for N_0, N_1, T and. At least two independent runs were performed for each sample with a total number of iterations always larger than 4×10^{10} steps. Different sets of priors were used to test their influence on the posteriors, but in most of the runs we set prior means for N_0, N_1, T (on a $\log_{10}$ scale) with means 4.0, 4.0 and 5.0, respectively; varying the standard deviations between one and five. For μ we set a mean of -3.5 with standard deviation of 0.25, so that values for the mutation rate in the region 10^{-4} to 10^{-3} had reasonable support, as widely assumed in demographic analysis.

For both the Beaumont[1] and Storz and Beaumont[2] methods, the first 10% of each independent analysis were discarded to avoid influence in parameter estimation by starting conditions (burn-in period). Convergence of the different chains was visually controlled and tested using Geweke[3] convergence diagnostic. The results of all runs were then pooled into one dataset in order to produce larger and more precise samples of the posterior distributions.

We also evaluated the strength of evidence for population expansion versus decline by calculating the Bayes factor[4] for each of the models as described by Storz and Beaumont[2] and Girod et al.[5]. In practice, the weights of evidence of the hypothesis that time is <800 years vs. >800 years, were assessed using approximate "Bayes factors" (BF), *i.e.* the ratio of the posterior densities of the two alternative hypotheses, over the ratio of the prior densities of the same two alternative hypotheses. BF ≥10 indicates strong support, BF between 3-10 indicates substantial support and BF ≤3 indicates no support. We identified hypotheses corresponding to the two factors as mentioned in the introduction that may have led to a population size decline in Bornean elephants. The two corresponding hypotheses were: (i) H1: the decline is recent and attributable to the recent introduction of elephants to Borneo (RI), within last 800 years, (iii) H2: the decline commenced following major climatic changes, during the last glacial maximum (20-18,000 thousand years ago (kya) but before the recent introduction of elephants, i.e. between 20-0.8 kya. BFs were first computed for each of the two time intervals against all other periods taken together as in Olivieri et al.[6] or Quéméré et al.[7]. Since the different hypotheses correspond to time periods of variable sizes, this may bias the results towards one hypothesis over the others. Thus, we also

performed a BF analysis by computing BFs for successive 500 years period as in Sharma et al.[8] and Quéméré et al.[7].

1.2 Further MSVAR results

Quantifying changes in effective population size and dating within each population

Using the Beaumont method[1], we found that each of the Borneo elephant populations exhibited a signal for population decline. The median $\log_{10}(r)$ was between -1.69 and -2.94 across all populations. This indicates a decline in the effective size of all populations of about two to three orders of magnitude and no support for stationarity ($\log_{10}(r) = 0$) or expansion ($\log_{10}(r) > 0$). The posterior distributions of the effective population size change for all elephant populations are shown in Fig. S1a.

Similarly, the Storz and Beaumont[2] method also suggested a strong decline in each of the Bornean elephant population. As shown in Fig. S1b, the data support a model in which the ancestral effective size (N_1) was larger than today (N_0). The estimates of current (median N_0) and ancestral population sizes (median N_1) were similar between the populations, and respectively ranged from 9 (Tabin) to 156 (Ulu Segama-Malua) and from 1,737 (Deramakot) to 7,176 (Ulu Segama-Malua).

The time since the onset of decline (T) exhibited a very large variance and differed between populations (Fig. S1c, and Supplementary Table S3). For most populations, the median T values were between 1,140 and 24,000 hence suggesting an ancient event. While our results indicate a more recent decrease in the Lower Kinabatangan and Deramakot (North Kinabatangan region), the decline appears to be older and prehistorical at all other sites including the sites in Central Sabah (i.e.,

Gunung Rara, Kalabakan, Ulu Segama-Malua) and Tabin Wildlife Reserve. This is confirmed by the Bayes Factor (BF) analysis (Fig. S2), which favored the H2 hypothesis that corresponds to a decline starting between 800 and 20,000 years BP. The test of the two hypotheses using the refined BF analysis of *T* posteriors favored the second (H2) (H2, BF > 2.27 – 7.97) over the first model (H1) (0.32 < BF < 2.3). In agreement with this result, the curves representing the BFs computed for 500 year periods for all sampled populations also shows low BF values for the recent past that increase for values greater than 800 years. However, we must also note that there are differences across the populations, either the posterior distributions of *T*, or in the BFs.

2. Approximate Bayesian computation (ABC) analyses

2.1 Details on the main demographic models

The demographic parameter priors used in ABC analyses were defined based on the current knowledge on Borneo elephants. We initially considered five simple (non-structured) demographic models, which differed in the date of introduction and on whether founder events occurred (Fig. 2): (i) *instantaneous decline* (ID) - large ancestral population that underwent instantaneous decline to their current stable effective size without a founder event, (ii) *exponential decline* (ED) - same as ID model but the decline is exponential rather than instantaneous, (iii) *ancient colonization* (AC) - large ancestral population decreased as a result of an ancient founder event that grows exponentially to the present-day population, (iv) *recent introduction from Sulu/Java* (RI) - introduction from Sulu/Java with a recent founder event following which the population experienced a rapid demographic expansion and (v) *two introductions* (TI) -

a combination of AC and RI models, incorporating two distinct introduction events; one "ancient" from mainland to Java and another "recent" from Java to Borneo. For all parameters, we chose to use priors over a wide range of biologically plausible values so that the choice of prior would have minimal influence on our estimates. For instance, in the ID and ED models, prior distributions on the timing of the decline (*T_shrink*) were wide enough to accommodate the possibility that reduction in population size resulted from climate change during the last glaciation maximum (LGM) events (15,000 - 22,500 years before present (YBP) of late Pleistocene when land bridges linked the Sunda islands and mainland[9,10]. Also, in the AC model, priors on the timing of the founding event included LGM. Demographic information for *E. maximus* indicates a generation time of 15 years[11]. Hence, *T_shrink* was set between 1,000 to 1,500 generations looking backwards in time in the AC model. In the RI model, prior values for *T_shrink* were 20 - 70 generations, to investigate recent events from 300 to 1000 years in the past[12,13]. As scant information is available on the likely effective population sizes of Bornean elephant, broad priors also were used. The priors for ancestral effective population size (*N_Anc*) were set large and wide (bounded between 10,000 and 100,000 with a loguniform distribution) considering that the size of ancestral mainland elephant population was large in the past. Generally, the effective size of populations, (*N_Cur*), is much smaller than the census size because of age structure, uneven sex-ratio etc. Also, according to a recent field survey in 2010, the numbers of elephants in Sabah were estimated at 2000[14]. Hence, the prior used for *N_Cur* was small for all models and was bounded between 251 and 1,000 with a loguniform distribution. The prior distributions of historical, demographic and mutational parameters are described in Table 3. We also inspected the posterior probability curves to check if the model fitting

could be improved by changing the ranges of priors. In several cases we updated prior ranges and ran particular scenarios one more time. As there is no existing knowledge on the mutation rate of microsatellite in elephants, we made the assumption that microsatellite loci followed a generalized stepwise-mutation model. We assumed a uniform mutation rate prior bounded between 10^{-5} and 10^{-3} for all loci.

In summary, all demographic models were specified by four demographical variable parameters: the ancestral effective population size (*N_Anc*), current effective population size (*N_Cur*) which is assumed to be same in all models, the effective number of founders (*N_ shrink*), and the time of bottleneck (*T_shrink*). In addition, as no more than two elephants were assumed to be part of the introduction founder event in late 13th century[12], we also defined a uniform prior (for *N_shrink*) bounded by 2 and 50 for the number of introduced elephants in the RI and TI models.

2.2. Structured models

Because the current elephant population in Borneo is geographically structured and because genetic data suggest that it is possibly comprised of two genetic clusters identified through Bayesian clustering analysis[15], we also tested two additional complex (structured) models introducing a subdivided population undergoing alternative possible demographic histories (Fig. S3): (i) *ancient colonization-split* (ACS)- a small population founded Borneo, expanded to a large population, then split into populations which all exponentially expanded into stable populations, (ii) *Recent introduction from Sulu/Java-split* (RIS)- same as model above with recent time (*T_shrink*) of introduction. All other historical and demographic parameters were kept same for these models. We assumed symmetric bidirectional migration in these three structured models, and the ranges of

priors were set to cover small level of gene flow (up to m=0.01). Both models were simulated with 8 demes. We have used identical migration between all demes at a rate m'=0.01, being m'=m/(D-1).

2.3 Summary statistics

For all ABC analysis, we used the mean and standard deviation (calculated over loci) for three summary statistics: number of alleles (*k*), expected heterozygosity (*He*[16]), allelic size range (*R*), thus a total of six statistics for model comparison. For the models with population structure, we added F_{ST}[17] as a metric of differentiation between populations. However, we did not use F_{ST} when comparing all five simple models with the two complex models with gene flow.

2.4 Validation of the model selection

We performed cross-validation analyses to evaluate if our ABC approach can distinguish between the five main demographic models. For that we took randomly 1,000 datasets from the simulation output for each of these models and used the function *cv4postpr* from the "abc" R package[18] to assign each of these datasets to a model. Cross-validation steps were performed thrice. First by comparing all five models against each other simultaneously, second pair-wise comparisons of all models, third comparing only the two models AC and RI (with structure; i.e. ACS and RIS) against each other. Results are given in the Supplementary Table S2 and are explained in the manuscript.

2.5 Goodness-of-fit

Prior to parameter estimation, it is important to check if the preferred model provides a good fit to the data i.e. is capable of reproducing the observed summary statistics from the real data. We assessed the goodness of fit for each demographic model using the *gfit* function of the "*abc*" R package[18] with 1,000 replicates, tolerance of 0.1 and the mean of the distance between observed and accepted summary statistics. In addition to this hypothesis-testing procedure, we also used *gfitpca* function of the "*abc*" R package[18] to build a PCA as a mean to visualize the fit between simulated and observed data sets. The function performs PCA using the *a priori* simulated summary statistics.

Compared with the 5 other simple demographic scenarios tested, only the datasets simulated under the AC scenario were compatible (p-value=0.90) with the observed summary statistics computed from the real data (Fig. S4). We also note that the *AC model with extended priors* was also able to reproduce the observed summary statistics of the elephant data (p-value=0.86). This finding hence provides further support to the very high posterior probability values obtained for AC model using the model choice procedure. Further, the PCA using the *a priori* simulated summary statistics suggests that all competing scenarios were capable of reproducing the observed summary statistics computed from the real data (Fig. S5).

3. *Supplementary ABC simulations*

In addition to the above simulations, supplementary ABC simulations were performed to explore the influence of prior assumptions about mutation rate, time of bottleneck on posterior estimates. A total of 100,000 simulations were run for each of these additional simulations. Models were compared by estimating their posterior probabilities using the

multinomial logistic regression approach on the simulations that are within 0.01 tolerance[19,20].

3.1 AC model with extended priors

For our initial simulations, we chose prior distributions tightly bounded around values based on the available historical data. However, to explore sensitivity to these assumptions, we simulated a new version of AC model with fixed mutation rate (10^{-3}) and wider priors on *N_Anc* and *T_shrink.* The prior on *N_Anc* was bounded between 1,000 and 100,000 individuals with a loguniform distribution. We set a uniform prior for *T_shrink* bounded between 20 and 1,500 generations, which corresponds to last 300 - 22,500 years. This prior range encompasses both the recent and ancient colonization time. For other population parameters such as current effective population size (*N_Cur*), and number of founders (*N_shrink*), same prior values were used as in the main demographic models. Model comparison showed that this version of AC model with fixed mutation rate value and wider priors on *N_Anc* and *T_shrink* has more posterior support than the original AC model (with wide prior on mutation rate). This model received a posterior probability of 0.56 whereas the original AC model had a posterior probability of 0.43. However, broader prior assumptions on *N_Anc* and *T_shrink* yielded smaller posterior estimates for ancestral population size coupled with smaller posterior estimates for time of bottleneck. The time of bottleneck (*T_shrink*) in this model seems to provide no support for values above 757 generations (that would correspond to around 11,400 years) and thus favours a LGM bottleneck scenario. The prior and posterior distributions of time and *N*e parameters from this simulation are available as joint plot in Fig. S6 and are listed in the Supplementary Table S4.

3.2 Effect of mutation rate

As mutation rate priors are likely to have a strong influence on estimates of effective population size (*N*e) and the time of bottleneck (*T_shrink*), AC and RI models using three different mutation rate values for microsatellite repeats were also tested and parameters were estimated. These include a low (10^{-5}), a medium (10^{-4}) and a high (10^{-3}) mean mutation rate over loci. For other population parameters, such as current and ancestral effective population sizes (*N_Cur* and *N_Anc*), number of founders (*N_shrink*) and *T_shrink* (time of bottleneck), same prior values were used as in the main demographic models.

Simulation of the two demographic models (AC and RI), a second time using different values of mutation rate still favoured the AC model (with mean mutation rate 10^{-3} for all loci) over RI. These additional simulations revealed that adjusting the prior on mutation rate parameter did have little effect on the other parameters of interest (i.e. *N_Anc, N_Cur* and *T_shrink*; see Fig. S7). Parameter estimates obtained for simulations exploring prior boundaries on mutation rate were in general highly congruent. Timing of decline was estimated at 18,000 years before present. These data hence support our main results that the founder event took place several thousand years ago in the history of Bornean elephant.

*3.3 AC model with equal priors on N*e

We have also simulated a modified version of AC model with equal and broader priors on *N*e. In all other models as described above, the priors do not overlap so a bottleneck was actually forced. Hence, we tested this model with same priors on *N*e to check if we still get a signature of the bottleneck. We used a loguniform prior for *N_Anc*, *N_Cur*

and *N_shrink* bounded between 100 and 100,000 individuals. The model comparison gave very strong support to this version of AC scenario (posterior probability close to 0.90). However, the posteriors are weakly defined and are wider, but sill supporting the same trend as in the original results: Lower *N_Cur* than *N_Anc*. Broader prior assumptions on *N*e yielded larger posterior estimates for *N_Anc* coupled (mean=8,709 [95% HPD: (331 – 79,432)]) with smaller posterior estimates for *N_Cur* (mean=1,096 [95% HPD: (117 – 25,118)]). While mode estimates were slightly different and gave (only) slightly larger values for *N_Anc*, posterior distribution of other parameters were overlapping among the two models (i.e., this version of AC model with broader priors and original AC model as described in the manuscript). Notably, the time (*T_shrink*) in this model also exhibits a posterior, which is again different from the prior and has a mean of 18,630 years before present.

3.4 MSVAR- like model

We also tested an ABC model emulating MSVAR model. For current (*N_Cur*) and ancestral effective population size (*N_Anc*), same prior values were used as in the main demographic model (the AC model). Model selection results gave no support (posterior probability= 0.0006) to this model. Hence this model was discarded.

Table S1. Model comparison using marginal densities, probabilities (low *p* value indicates an inability of the model to produce the summary statistics).

Model	Marginal density	*P* value
instantaneous decline (ID)	1,21E-04	0,005
exponential decline (ED)	1,61E-10	0
ancient colonization (AC)	1,06E-01	**0,67**
recent introduction from Sulu/Java (RI)	4,31E-06	0,001
two introductions (TI)	7,96E-05	0,006
ancient colonization-split (ACS)	1,71E-01	**0,84**
recent introduction from Sulu/Java-split (RIS)	7,51E-07	0

Table S2. Model choice cross-validation.

Confusion matrices for a) all the five simple models studied, b) AC model against each of the other simple models and c) comparing the AC and RI models and their equivalents with structure against each other. The model from which each dataset was simulated is shown in the row label (true model), while the model identified as best by the model choice algorithm is shown in the column label (estimated model). "Best" models were those with the highest posterior probability. Cell values indicate the proportion of simulations (in percentages) from each true model that were chosen for a given estimated model (each row sums to 100) considered.

a)

Model	ID	ED	AC	RI	TI
instantaneous decline (ID)	24,2	9,6	28,9	19,2	18,1
exponential decline (ED)	6,1	61,6	5,4	16,6	10,3
ancient colonization (AC)	7,2	2,6	73,6	10	6,6
recent introduction from Sulu/Java (RI)	9,5	21,4	27,6	32,4	9,1
two introductions (TI)	13,6	12,7	33,6	17,2	22,9

b)

Model		*X*	ancient colonization (AC)
x=ID	X	91	9
	AC	6	94
x=ED	X	98	2
	AC	2	98
x=RI	X	83	17
	AC	8	92
x=TI	X	94	5
	AC	6	95

where x is the respective model

c)

Model	AC	ACS	RI	RIS
ancient colonization (AC)	41.3	46.9	9.1	2.7
ancient colonization-split (ACS)	33.2	59.5	5.5	1.8
recent introduction from Sulu/Java (RI)	10.3	6.1	45.1	38.5
recent introduction from Sulu/Java-split (RIS)	3.1	4.3	19.9	72.7

Table S3. Posterior values of the bottleneck signal obtained with MSVAR1.3. Parameters values have been transformed from log to linear scale.

Elephant populations analyzed	Demographic parameters	Lower Bound	Mean	Median	Upper bound
Lower Kinabatangan	N_0	0.28	34	54	836
	N_1	646	3,332	2,617	46,708
	T	8	1,373	1,140	346,559
Tabin	N_0	0.07	6	9	96
	N_1	843	7,066	7,103	58,018
	T	22	1,566	2,260	33,573
Gunung Rara	N_0	2	45	64	316
	N_1	475	3,016	2,862	22,566
	T	122	6,015	8,050	89,508
Deramakot	N_0	0.14	10	14	166
	N_1	521	1,741	1,737	5,840
	T	10	435	574	6,745
Kalabakan	N_0	1	40	57	291
	N_1	767	4,103	3,860	26,792
	T	137	5,559	7,452	72,702
Ulu Segama-Malua	N_0	38	148	156	511
	N_1	1,441	7,333	7,176	39,958
	T	3,953	22,784	24,109	117,068
Pooled individuals	N_0	143	796	833	4,766
	N_1	753	5,317	4,820	53,154
	T	1,028	50,488	57,056	1,311,243

Table S4. *Ancient colonization* (*AC*) *model with extended priors*: point estimates and 95% credibility intervals for all parameters obtained through simulations evoking different prior assumption on *T_shrink and N_Anc*. Note: posterior estimate values were converted from log to linear scale.

Parameter	Description	Prior	Posterior				
		type [min,max]	5%	Mean	median	mode	95% HPD
N_Anc	ancestral effective population size	loguniform [3,5]	1,071	7,863	6,936	1,892	93,239
N_Cur	current effective population size	loguniform [2.4,3]	454	718	730	751	982
T_shrink *(in generations)*	time of bottleneck	uniform [20,1500]	273	757	718	576	1,374
N_shrink	effective number of individuals introduced	uniform [2,50]	4	21	19	9	46

Table S5. Results from the simulations using mtDNA sequence data. 100,000 simulations were done under each model and the proportion of simulations with zero diversity for each model were counted.

Model	Number of simulations with zero summary statistics
instantaneous decline (ID)	46,161
exponential decline (ED)	12,748
ancient colonization (AC)	64,344
recent introduction from Sulu/Java (RI)	49,451
two introductions (TI)	54,564

Figure S1. MSVAR estimates of changes in effective population size in Bornean elephant population. This is shown here for each of the investigated site in Sabah; a) Posterior distributions of the effective population size change, log (N_0/N_1) using the exponential population size change model. Log (N_0/N_1) represents the ratio of present (N_0) to past (N_1) population size. The vertical dashed line corresponds to absence of population size change, log (N_0/N_1)=0. The prior distribution is shown for comparison (flat dotted line), b) Posterior distributions for the past (N_1) and present (N_0) effective population sizes using MSVAR 1.3. Dashed lines correspond to the different priors used for N_0 and N_1, c) Posterior distributions for the time since elephant populations collapse in years (T) represented in $\log_{10}$. For all the figures, the solid lines correspond to the posterior distributions obtained by multiple independent runs. In figure S1 a) and c) the vertical black dotted line corresponds to 95% quantile of the posterior distribution.

a)

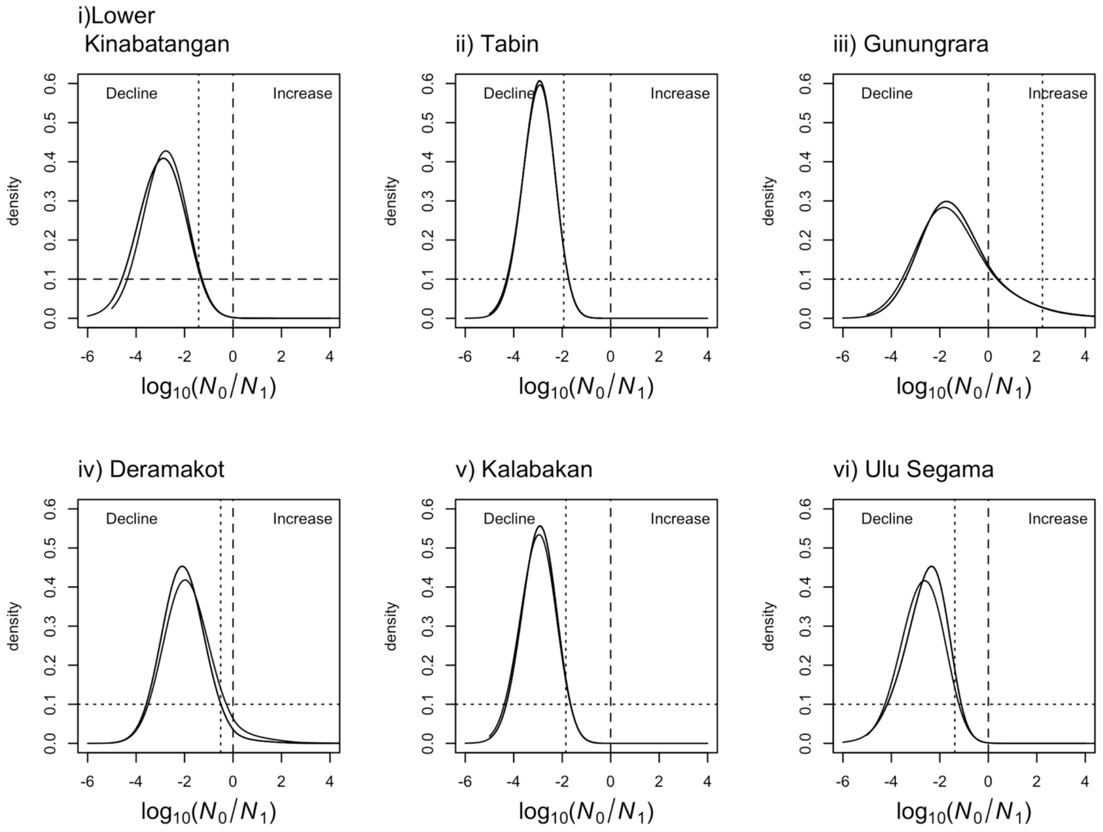

b)

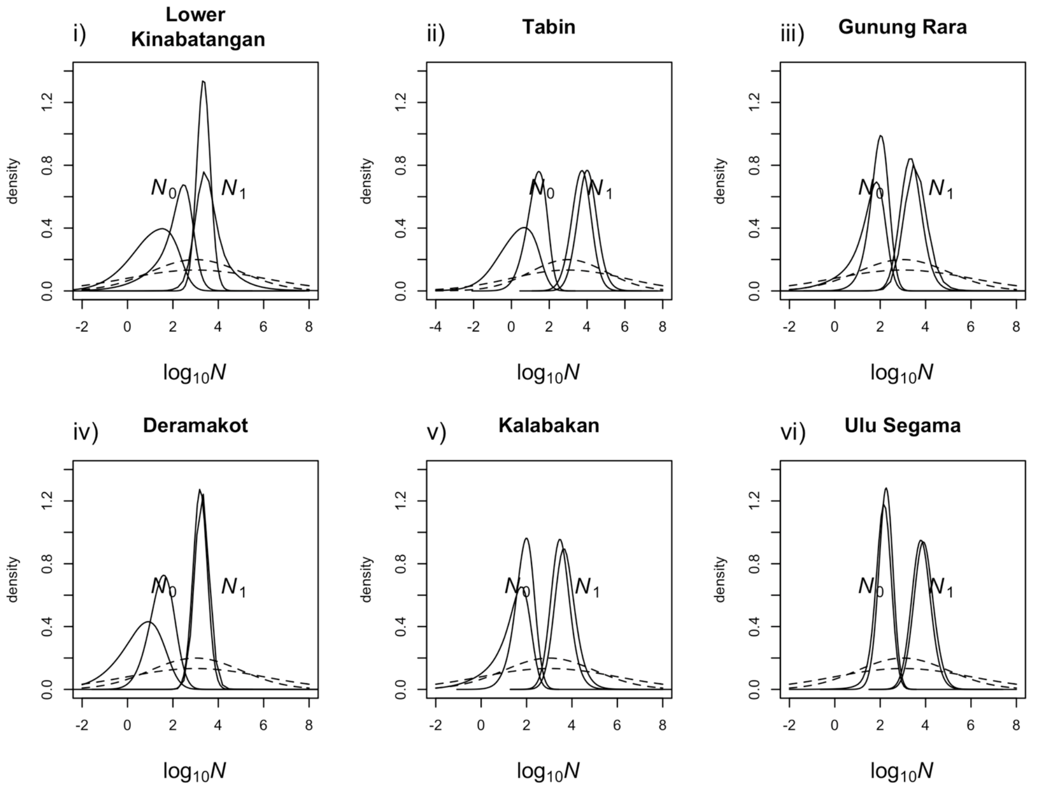
i)
Lower Kinabatangan
ii)
Tabin
iii)
Gunung Rara
iv)
Deramakot
v)
Kalabakan
vi)
Ulu Segama
density
N_0
N_1
$\log_{10}N$

c)

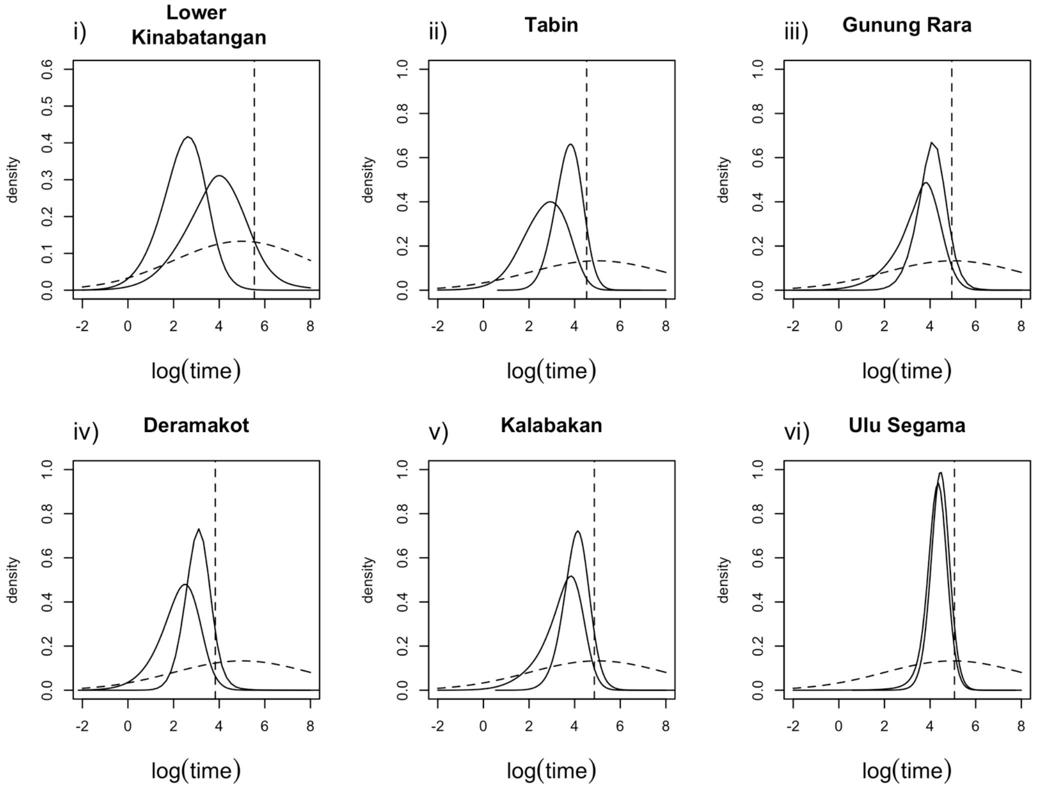
i)
Lower Kinabatangan
ii)
Tabin
iii)
Gunung Rara
iv)
Deramakot
v)
Kalabakan
vi)
Ulu Segama
density
log(time)

Figure S2. Bayes' factors (BFs) for the time of the beginning of the demographic changes (*T*) calculated for the Borneo elephant in the MSVAR analyses. Results correspond to the population-level analyses. BFs >4 are considered as 'positive evidences', while BFs >7 are considered as significant. a) The BF values of parameter *T* (time since the beginning of the population collapse) for all elephant populations were computed for each 500 year time steps and are plotted for last 20,000 years. The BF value of 4 is plotted as horizontal dash line. The vertical dash lines correspond to the dates of recent introduction ~ 0.8 kya, and last glacial maximum ~ 18 kya. b) H1 and H2 represent two different hypotheses tested. Phase H1 is most recent and corresponds to the recent introduction of elephants to Borneo within last 800 years, Phase H2 corresponds to major climatic changes during the last glacial maximum.

a)

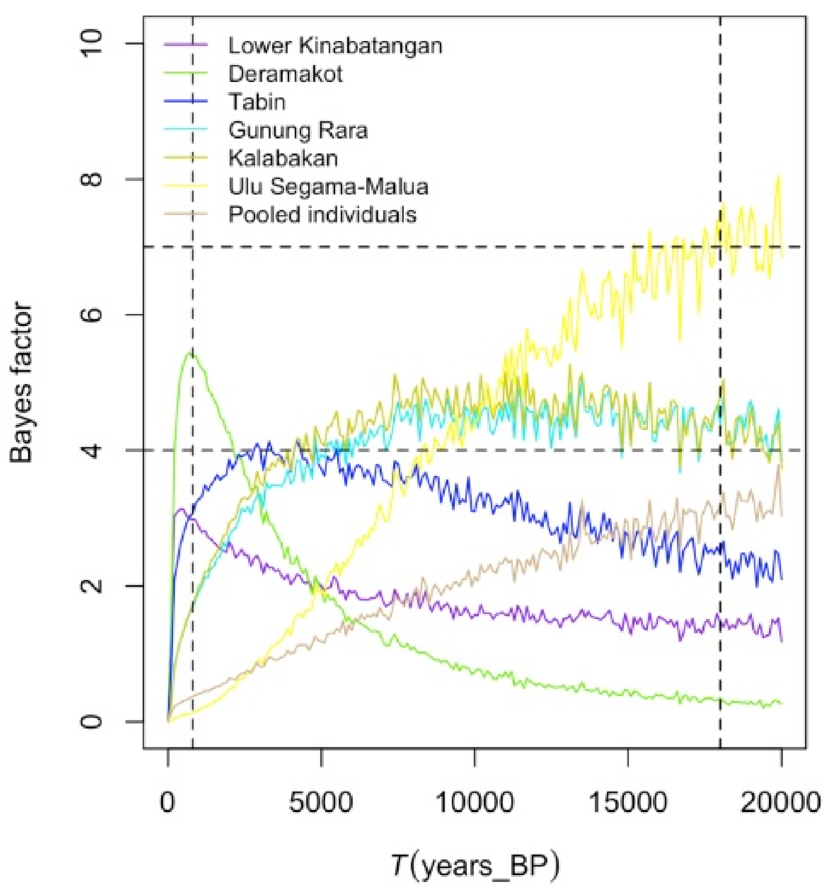

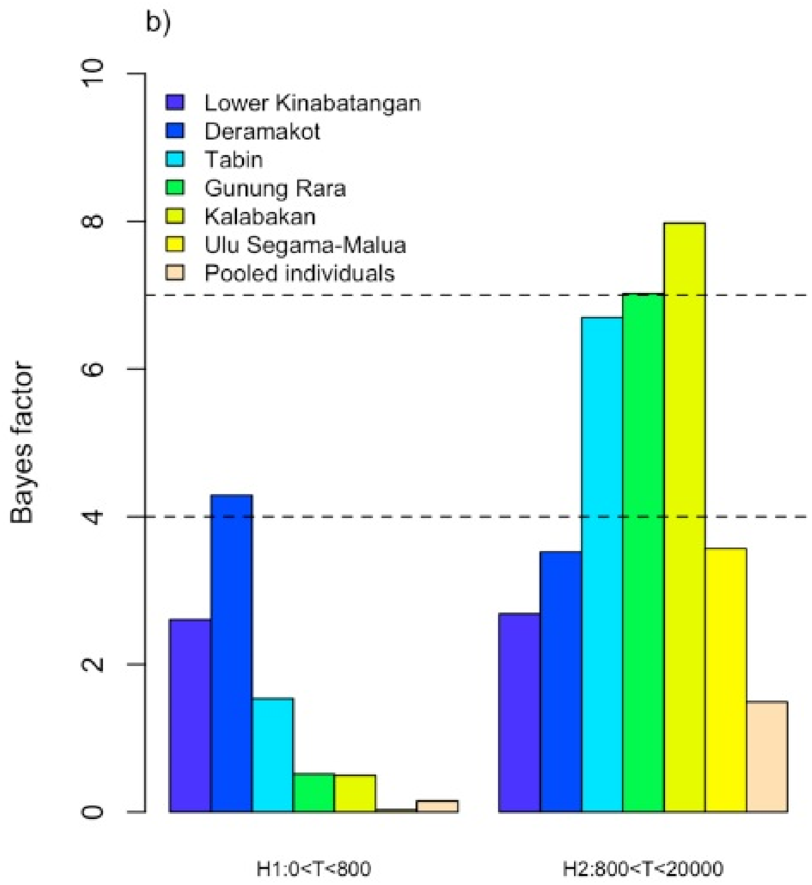
b)
Lower Kinabatangan
Deramakot
Tabin
Gunung Rara
Kalabakan
Ulu Segama-Malua
Pooled individuals
Bayes factor
0
2
4
6
8
10
H1:0<T<800
H2:800<T<20000

Figure S3. *Ancient colonization split* (ACS) and *recent introduction split* (RIS) model with 8 demes (denoted d1 ... d8) of haploid size *N_Cur*. The migration rate m´=m/(D-1) is identical between all demes.

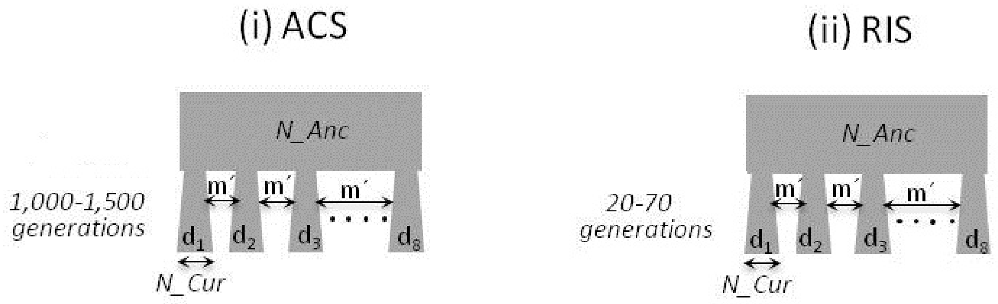

Figure S4: Histogram of the null distribution of the test statistic for goodness-of-fit assuming the *ancient colonization* (AC) scenario, which was selected on the ABC model choice procedure.

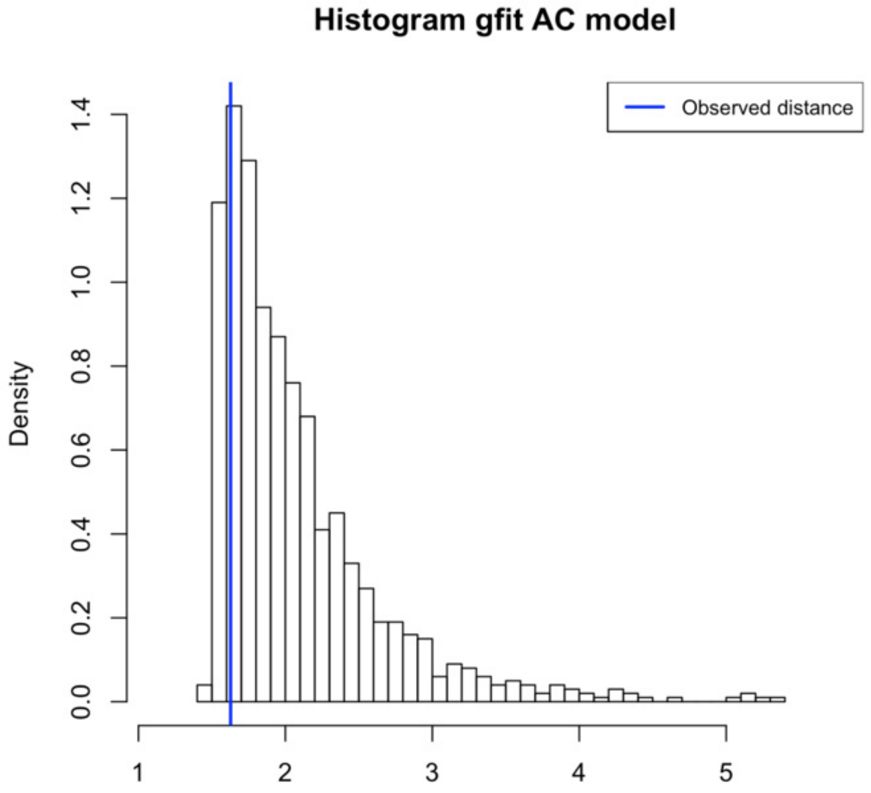

Figure S5: 90% envelope of the 2 Principal Components obtained with each demographic model. Simulations were used *a priori* and the cross corresponds to the observed value.

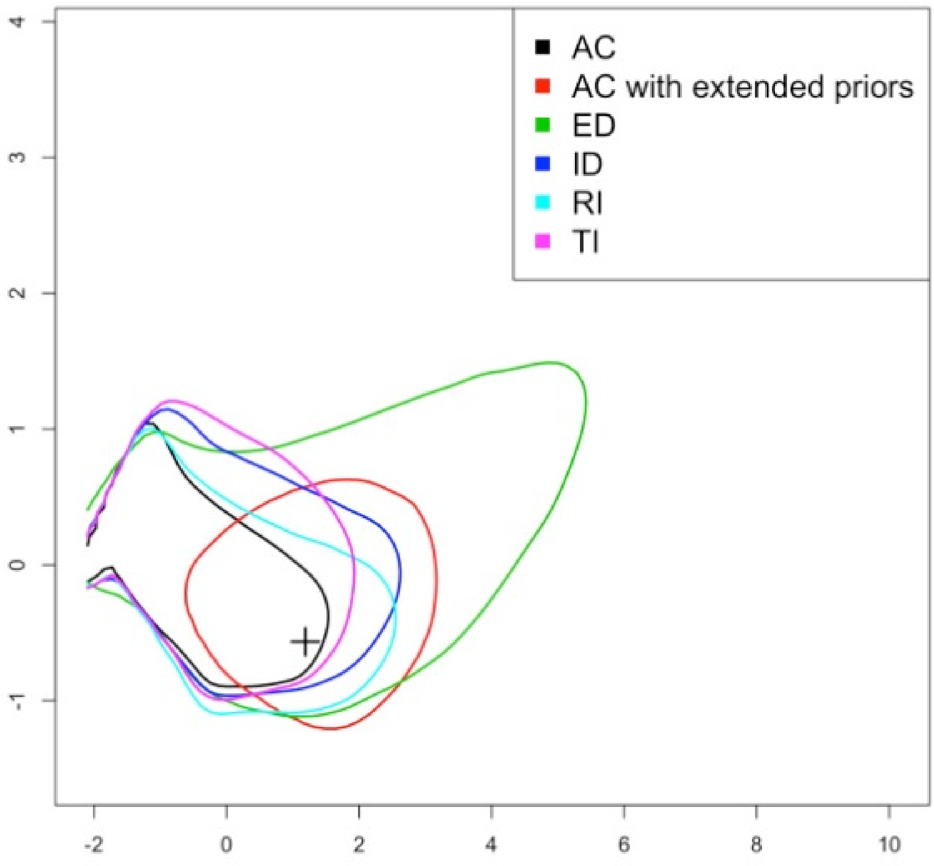

Figure S6. ABC posterior parameter density estimates of the *ancient colonization* (AC) model under broader prior assumptions on *T_shrink* and *N_Anc* (see supporting materials for details). Horizontal grey lines represent the prior distribution, and black thick line represents posterior distribution of the model. Point estimates and 95% credibility intervals for all key parameters obtained through simulations are also given. Note that in a) and b) values were converted from log to linear scale.

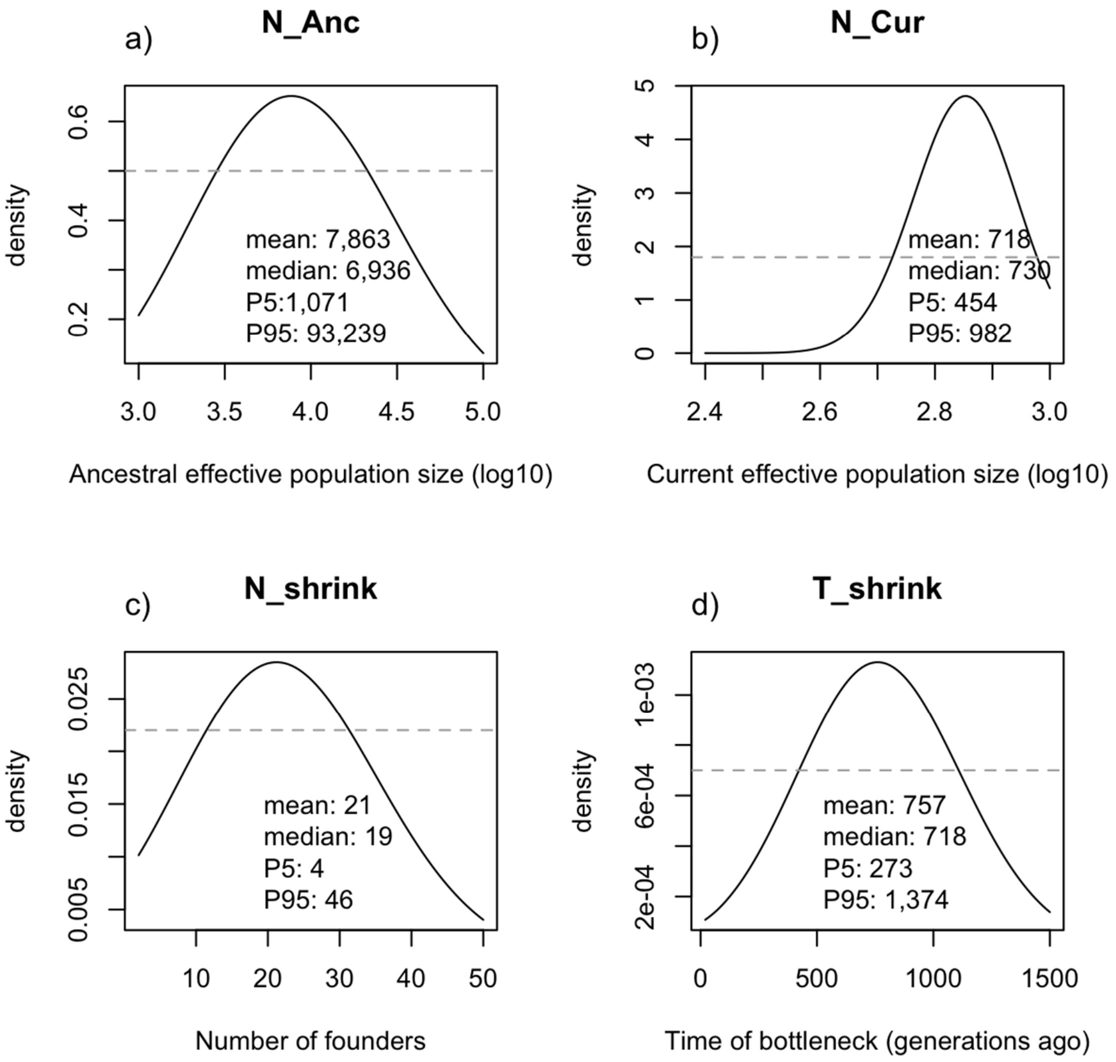

Figure S7. ABC posterior parameter density estimates and effect of the microsatellite mutation rate prior specification. Black thick line represents posterior distribution of the *ancient colonization* (AC) model with wide priors on mutation rate (10^{-5} to 10^{-3}) for all microsatellite loci. Purple thick line represents posterior distribution of the AC model with fixed mutation rate (10^{-3}). Horizontal grey lines represent the prior distribution, and the thick lines represented the posterior distribution.

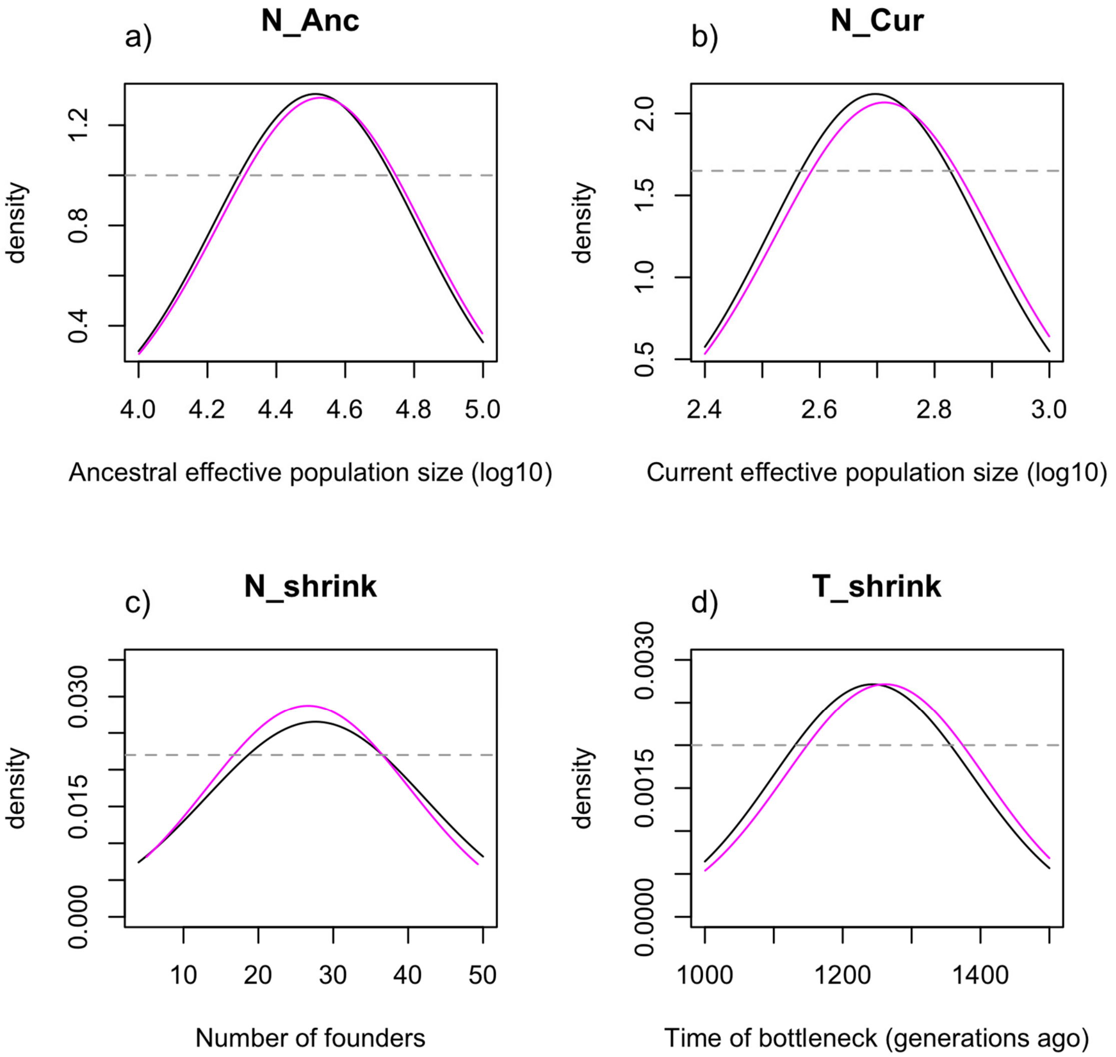

Figure S8. Plot of allelic richness averaged for the simulated models (*ancient colonization* and *recent introduction from Sulu/ Java*) compared to the allelic richness averaged over 18 microsatellite loci in contemporary Bornean elephant population (n = 224)[15].

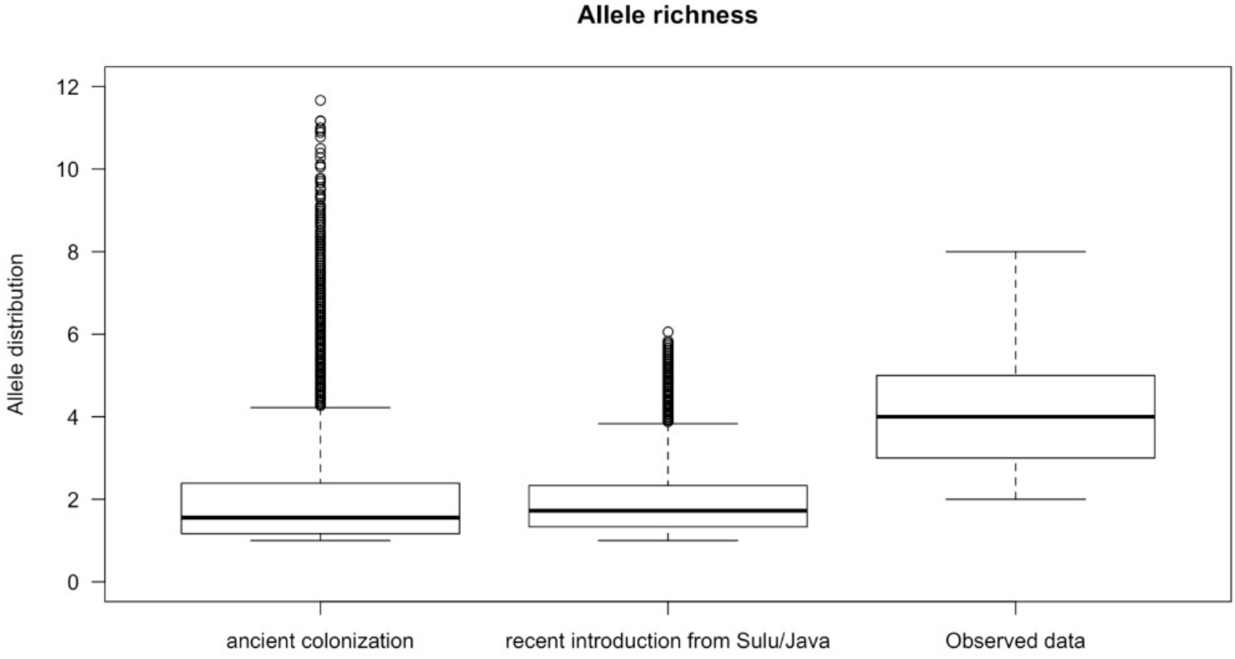

References

1. *Beaumont, M.A. Detecting population expansion and decline using microsatellites. Genetics 153, 2013–2029 (1999).*
2. *Storz, J.F. and Beaumont, M.A. Testing for genetic evidence of population expansion and contraction: an empirical analysis of microsatellite DNA variation using a hierarchical bayesian model. Evolution 56, 154–166 (2002).*
3. *Geweke, J. Evaluating the accuracy of sampling-based approaches to the calculation of posterior moments. In Proceedings of the Fourth Valencia International. Meeting on Bayesian Statistics, Oxford University Press 169–193 (1992).*
4. *Kass, R.E. and Raftery, A.E. Bayes factors. Journal of the American Statistical Association 90, 773–795 (1995).*
5. *Girod, C., Vitalis, R., Lebois, R., Freville, H. Inferring population decline and expansion from microsatellite data: a simulation-based evaluation of the MSVAR methods. Genetics 188, 165–179 (2011).*
6. *Olivieri, G.L., Sousa, V., Chikhi, L., Radespiel, U. From genetic diversity and structure to conservation: Genetic signature of recent population declines in three mouse lemur species (Microcebus spp.). Biological Conservation 141, 1257–1271 (2008).*
7. *Quéméré, E. et al. Genetic data suggest a natural prehuman origin of open habitats in northern Madagascar and question the deforestation narrative in this region. Proceedings of the National Academy of Sciences 109, 13028-13033 (2012).*
8. *Sharma, R. et al. Effective population size dynamics and the demographic collapse of Bornean orang-utans. PLoS ONE 7 (2012).*
9. *Voris, H.K. Maps of Pleistocene sea levels in Southeast Asia: shorelines, river systems and time durations. Journal of Biogeography 27, 1153–1167 (2000).*
10. *Bird, M.I., Taylor, D., Hunt, C. Environments of insular Southeast Asia during the last glacial period: a savanna corridor in Sundaland? Quaternary Science Reviews 24, 2228 – 2242 (2005).*
11. *Sukumar, R. The Asian Elephant: Ecology and Management. Cambridge University Press, Cambridge (1989).*
12. *Shim, P.S. Another look at the Borneo elephant. Sabah Society Journal. 20, 7-14 (2003).*
13. *Cranbrook, E. O., Payne, J., Leh, C.M.U. Origin of the elephants Elephas maximus L. of Borneo. Sarawak Museum journal 63, 95-125 (2008).*
14. *Alfred, R., Ahmad, A.H., Payne, J., Williams, C., Ambu, L. Density and population estimation of the Bornean elephants (Elephas maximus borneensis) in Sabah. OnLine Journal of Biological Sciences 10, 92-102 (2010).*
15. *Goossens, B. et al. Habitat fragmentation and genetic diversity in natural populations of the Bornean elephant: Implications for conservation. Biological Conservation 196, 80-92 (2016).*
16. *Nei M. Estimation of average heterozygosity and genetic distance from a small sample of individuals. Genetics 89, 583–590 (1978).*
17. *Weir, B.S. and Cockerham, C.C. Estimating F-statistics for the analysis of population structure. Evolution 38, 1358–1370 (1984).*
18. *Csilléry, K., Blum, M., Francois, O. abc: an R package for approximate Bayesian computation (ABC). Methods in Ecology and Evolution 3, 475–479 (2012).*
19. *Fagundes, N.J.R. et al. Statistical evaluation of alternative models of human evolution. Proceedings of the National Academy of Sciences of the United States of America 104, 17614-17619 (2007).*

20. *Beaumont, M. Joint determination of topology, divergence time, and immigration in population trees. In: Matsumura S, Forster P, Renfrew C, editors. Simulation, genetics, and human prehistory. Cambridge: McDonald Institute for Archaeological Research (2008).*
